# Associations between small and middle molecules clearance and the change of cognitive function in peritoneal dialysis

**DOI:** 10.1007/s40620-019-00661-8

**Published:** 2019-10-23

**Authors:** Yi Li, Hai-chen Pi, Zhi-Kai Yang, Jie Dong

**Affiliations:** 1 Renal Division, Department of Medicine, Peking University First Hospital; Institute of Nephrology, Peking University; Key Laboratory of Renal Disease, Ministry of Health; Key Laboratory of Renal Disease, Ministry of Education, Beijing, 100034 China; 2 Renal Division, Xinqiao Hospital, Army Medical University, Chongqing, China; 3 Emergency Department, Peking University First Hospital, Beijing, China

**Keywords:** Peritoneal dialysis, Urea, Creatinine, Beta-2 microglobulin, Solute, Clearance, Cognitive function, Executive function

## Abstract

**Background:**

Uremic toxins have been suspected as potential contributors for cognitive impairment in peritoneal dialysis (PD) patients. However, associations between the clearance of serum small and middle molecules and the change of cognitive function were not fully explored and then we explored this issue in the present study.

**Method:**

A total of clinically-stable 222 patients on PD were enrolled and then followed up for 2 years in this single-center prospective cohort study. Small and middle molecules clearances were examined by urea clearance (Kt/V), creatinine clearance (Ccr) and beta-2 microglobulin (B2M) clearance via dialysate and urine at baseline and after 2 years. Global and specific cognitive impairment were measured at baseline and after 2 years. Modified Mini-Mental State Examination (3MS) was assessed for global cognitive function, trail-making tests A and B for executive function and subtests of the battery for the assessment of neuropsychological status for immediate and delayed memory, visuospatial skills and language ability.

**Results:**

The median of total Kt/V, Ccr and B2M clearance were 1.89, 53.2 l/w/1.73 m^2^ and 17.5 l/w/1.73 m^2^, respectively at baseline. The prevalence of global cognitive impairment was 12.3% for 222 patients and 15.4% for the remained 130 patients after 2 years. At baseline, total Kt/V was independently positively associated with delayed memory function. Total and dialysate beta-2 microglobulin clearance was positively associated with 3MS scores and negatively with completion time on trail A after multivariate adjustment. At 2 years, we observed a significant difference in the changing trend of 3MS scores between groups divided by total B2M clearance (*P *= 0.033), which still maintained to be meaningful after multivariate adjustment (*P *= 0.024). Patients with total B2M clearance > 19.0 l/w/1.73 m^2^ got significant improvement on their 3MS scores (*P *= 0.005). Patients divided by total Kt/V or Ccr were not significantly different in the trends of general and any specific cognitive function during the follow up.

**Conclusion:**

The higher middle molecules clearance independently correlated to better performance on general cognitive and executive function in PD patients, which also predict an improvement in general cognitive function during the follow up.

**Electronic supplementary material:**

The online version of this article (10.1007/s40620-019-00661-8) contains supplementary material, which is available to authorized users.

## Introduction

Cognitive impairment (CI) has been shown to be an independent predictor of mortality [1] and technique survival [2] in patients on maintenance dialysis. Unfortunately, the prevalence of CI is high between 27% and 67% among patients on peritoneal dialysis (PD) or hemodialysis [1, 3–6]. To determine risk factors of CI and take measures for intervention is of importance in this population.

There are multifactorial mechanisms for the declined cognitive function of patients with chronic kidney disease (CKD), including age, socioeconomic and education status, cardiovascular risk factors and uremia- or dialysis-related factors [7–9]. Among them, uremic toxins are suspected to contribute to the aggravated cognitive dysfunction across the CKD stages since concentrations of small, middle and large molecules will inevitably increase with the loss of renal function. As shown in previous observational data, glomerular filtration rate was closely associated with cognitive function in CKD patients [10–12].

However, the relationship between urea clearance and cognitive function was not conclusive among hemodialysis patients [1, 13, 14]. Interventional studies focusing on the improvement of solute clearance, especially on small-molecule clearance, by increasing dialysis frequency or dialysis duration, on cognitive function showed inconsistent findings [15–17].

The beta-2 microglobin (B2M) is a marker of middle-molecule uremic toxin. The main concern with the high B2M concentration relates to its capacity to generate amyloid deposits that are mainly located in the musculoskeletal. In the past 10 years, more attentions have been paid on the relationship between serum B2M and cardiovascular risk and mortality [18]. Most recently, one study indicated that systemic B2M accumulation contribute to age-related cognitive dysfunction and impairs neurogenesis in aged mice [19]. It is of interest to know whether the concentrations of small- and middle-molecule uremic toxins, or their clearance levels via dialysate or urine could link to the CI in dialysis patients. In this study, we aimed to investigate this issue through this prospective observational study performed in peritoneal dialysis (PD).

## Materials and methods

### Study design and participants

This is a single-center prospective cohort study of cognitive function in PD patients. Totally 222 patients were enrolled from Peking University First Hospital between March 2013 and August 2013. We collected demographics data, comorbidities, laboratory data, and cognitive function assessment at baseline for all participants. All of them were prospectively followed. For whom still being maintained on PD between March 2015 and August 2015, we measured their cognitive function after 2 years. The ethics committee of Peking University First Hospital approved the study. Patients gave written consent for their information to be stored in the hospital database and for it to be used in research.

This study enrolled prevalent PD patients between March 2013 and August 2013. Inclusion criteria for participants were: age ≥ 18 years; had been undergoing PD ≥ 3 months and clinically stable; able to undergo all measurements and questionnaires as required. Patients were excluded if they had a systemic infection, acute cardiovascular events, active hepatitis or cancer, surgery or trauma in the month prior to the study, and all other study-obstructive conditions such as severe eyesight loss, language incompatibility, illiteracy, mental disturbance (preexisting dementia or confusion, and various mental disorders), upper limbs disability. All the subjects received conventional glucose-based, lactate-buffered PD solutions (Ultrabag; Baxter Healthcare, Guangzhou, China).

### Clinical characteristics

Demographics and comorbidities were recorded, including age, gender, education level, durations of PD, body mass index (BMI), systolic and diastolic blood pressure, primary kidney disease, the presence of diabetes mellitus (DM), and history of cardiovascular disease (CVD). Mean arterial pressure was calculated. Level of education was recorded as the highest school level at which a diploma was received, that is, elementary school or lower; middle school; high school; or above high school. CVD was recorded if one of the following conditions was present: angina, class III–IV congestive heart failure (NYHA), transient ischemic attack, history of myocardial infarction or cerebrovascular accident and peripheral arterial disease [20].

### Laboratory methods

After overnight fasting while continuing PD therapy, participants had their venous blood sampled for routine and biochemical measurements. Biochemical data including serum sodium, serum albumin, calcium and phosphate, triglyceride, total cholesterol, high-sensitivity C-reactive protein (hsCRP), and hemoglobin were calculated as the mean of measurements taken over the preceding 3 months. Biochemical profiles were investigated using an automatic Hitachi chemistry analyzer. Residual renal function (RRF) was defined as the mean of residual creatinine and urea clearance from collecting 24-h urine. Dialysis adequacy was defined as total Kt/V and creatinine clearance. The beta-2 microglobulin (B2M) in serum, dialysate, and urine was examined using a commercial radioimmunoassay kit (JD MBio Corp., TJ, China). Intra-assay and inter-assay variabilities were 4.7% and 10.8%, respectively. The B2M clearance via dialysate, urine and total was calculated respectively and normalized by body surface area.

### Cognitive function

The full battery consisted of the Modified Mini-Mental State Examination (3MS) [21] to test overall cognitive function. Global cognitive impairment was defined as a score of less than 80 in 3MS test in previously observational studies of cognitive function [5, 12, 22, 23]. Because mean scores on the 3MS vary by education, we used a 3MS cutpoint of < 75 for individuals with less than a high school education and a 3MS cutpoint of < 80 for individuals with a high school education [12].

Specific cognitive function was measured as executive function, i.e. trail making tests A (trail A) and B (trail B), immediate memory, delayed memory, visuospatial skill and language ability by subtests of repeatable battery for the assessment of neuropsychological status (RBANS). The trail A and trail B [24] were to test executive function including decision-making and processing speed. Executive dysfunction was defined as a trail A score more than 75 s and trail B more than 180 s [25–27]. In addition, subtests of RBANS was adopted to assess immediate memory (list learning and story memory), delayed memory (list recall, list recognition, story recall, and figure recall), visuospatial skill (figure copy), and language ability (picture naming and semantic fluency) respectively [28]. The reliability and validity of RBANS have been already proved in Shanghai and Beijing population [29, 30]. The raw scores were transferred to age-standardized T score for all subtests of RBANS. The T scores less than 1 SD below the published mean in education-grouped Chinese populations were identified as impaired for each test [31].

Assessments of cognitive function were performed by the same group at baseline and after 2 years in a separated room with one medical staff to one patient. Totally four medical staffs participated in this study as observers and they all completed a training program that taught them the methods and processes to ensure the integrity and accuracy of the assessments.

### Statistical analysis

Continuous data were presented as mean ± SD except for durations of PD, RRF, and hsCRP, which were presented as the median with interquartile range, due to high skew. Categorical variables were presented as proportions. Correlations of small and middle molecules clearance, and parameters of cognitive functions at baseline were examined by using univariable correlation analysis. Then each marker for solute clearance at baseline was examined with its independent relationship with cognitive functions by multivariable linear regression analysis adjusting for recognized confounders such as age, dialysis vintage, education level, the presence of diabetes, serum albumin, hs-CRP, hyponatremia and mean arterial pressure. For whom being maintain PD, cognitive function at baseline and 2-year later was compared by paired-sampled T tests. We further explored the changing trend of 3MS scores and executive function as assessed by completion time on trail A and trail B between groups according to total Kt/V and total B2M clearance by using a mixed model analysis. The baseline value of the outcome variables was adjusted as a model covariate. Sensitivity analysis was performed with adjustment of patients’ age, dialysis vintage, education level, the presence of diabetes, serum albumin, hs-CRP, hyponatremia and mean arterial pressure. Comparisons in 3MS scores and executive function as assessed by completion time on trail A and trail B in each group were also explored by paired-sampled T tests. Total Kt/V was divided into three groups: < 1.7, 1.7–2.0 and > 2.0. Total B2M clearance was divided into three groups according to tertiles.

All probabilities were two-tailed, and the level of significance was set at 0.05. Odds ratios (ORs) and 95% CI were calculated. Statistical analysis was performed using SPSS for Windows, software version 20.0 (SPSS Inc., Chicago, IL).

## Results

### Basic characteristics and follow up

A total of 278 patients were eligible for the study and 222 (79.9%) gave consent. All of them completed clinical datas and cognitive function assessments, with the mean age of 56.1 years, PD durations of 30.9 months, BMI of 23.1 kg/m^2^, hemoglobin of 113.8 g/l, and serum albumin of 38.9 g/l. Of these patients, 46.8% were men, 32.4% were diabetics, 29.3% had a history of CVD, and 65.8% had a diploma of high school or higher education level (Table 1).

**Table 1:** Clinical characteristics of PD patients

Variables	All patients	Remained patients at 2 year
Num	222	130
Age (years)	56.1 ± 12.8	55.7 ± 11.9
Male (%)	104 (46.8%)	57 (43.8%)
PD duration (months)	30.9 (13.2–58.8)	30.4 (14.2–56.3)
Diabetes (%)	72 (32.4%)	37 (28.5%)
Cardiovascular disease (%)	65 (29.3%)	35 (26.9%)
Level of education		
Elementary school or lower	17 (7.66%)	10(7.7%)
Middle school	59 (26.6%)	31(23.8%)
High school	81 (36.5%)	49(37.7%)
Above high school	65 (29.3%)	40(30.8%)
Body mass index (kg/m^2^)	23.1 ± 3.5	22.7 ± 3.4
Mean arterial pressure (mmHg)	94.1 ± 9.8	94.4 ± 10.1
Hemoglobin (g/l)	113.8 ± 10.9	114.9 ± 10.4
Serum albumin (g/l)	38.9 ± 3.2	39.4 ± 3.1
Triglyceride (mmol/l)	2.3 ± 1.4	2.3 ± 1.5
Total cholesterol (mmol/l)	4.7 ± 0.9	4.7 ± 1.0
Serum sodium (mmol/l)	139.4 ± 2.3	139.6 ± 2.1
Calcium (mmol/l)	2.4 ± 0.2	2.4 ± 0.2
Phosphate (mmol/l)	1.7 ± 0.4	1.6 ± 0.3
Hs-CRP (mg/l)	3.0 (0.9–8.9)	2.1 (0.7–6.7)

*PD* peritoneal dialysis, *hs-CRP* high-sensitivity C-reactive protein, *Kt/V* urea clearance per week, *Ccr* creatinine clearance, *B2M* beta-2 microglobulin

**Fig. 1:**
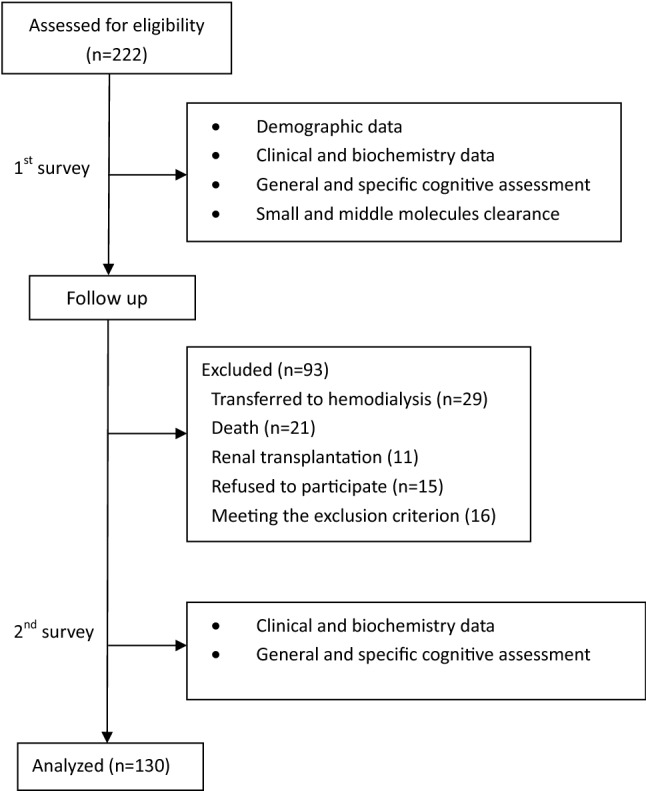
Flow chart of the study

During the follow-up, 29 patients were transferred to hemodialysis, 21 died, 11 received renal transplantation, 15 refused to participate the second cognitive assessments and 16 were excluded due to eyesight loss and acute comorbidities. Therefore, the remained 130 patients were examined with cognitive function repeatedly (Fig. 1). The mean albumin was significantly higher and hs-CRP was lower in the excluded 92 patients as compared to the remained 130 patients (*P *= 0.005 and 0.04, respectively). The delayed memory function was significantly worse in the excluded patients as compared to the remained patients (*P *= 0.02). The general cognitive function, executive function, immediate memory, language and visuospatial function were not significantly different between two group (*P *> 0.05).

### Concentration and clearance of small and middle molecules solute and cognitive function at baseline

**Fig. 2:**
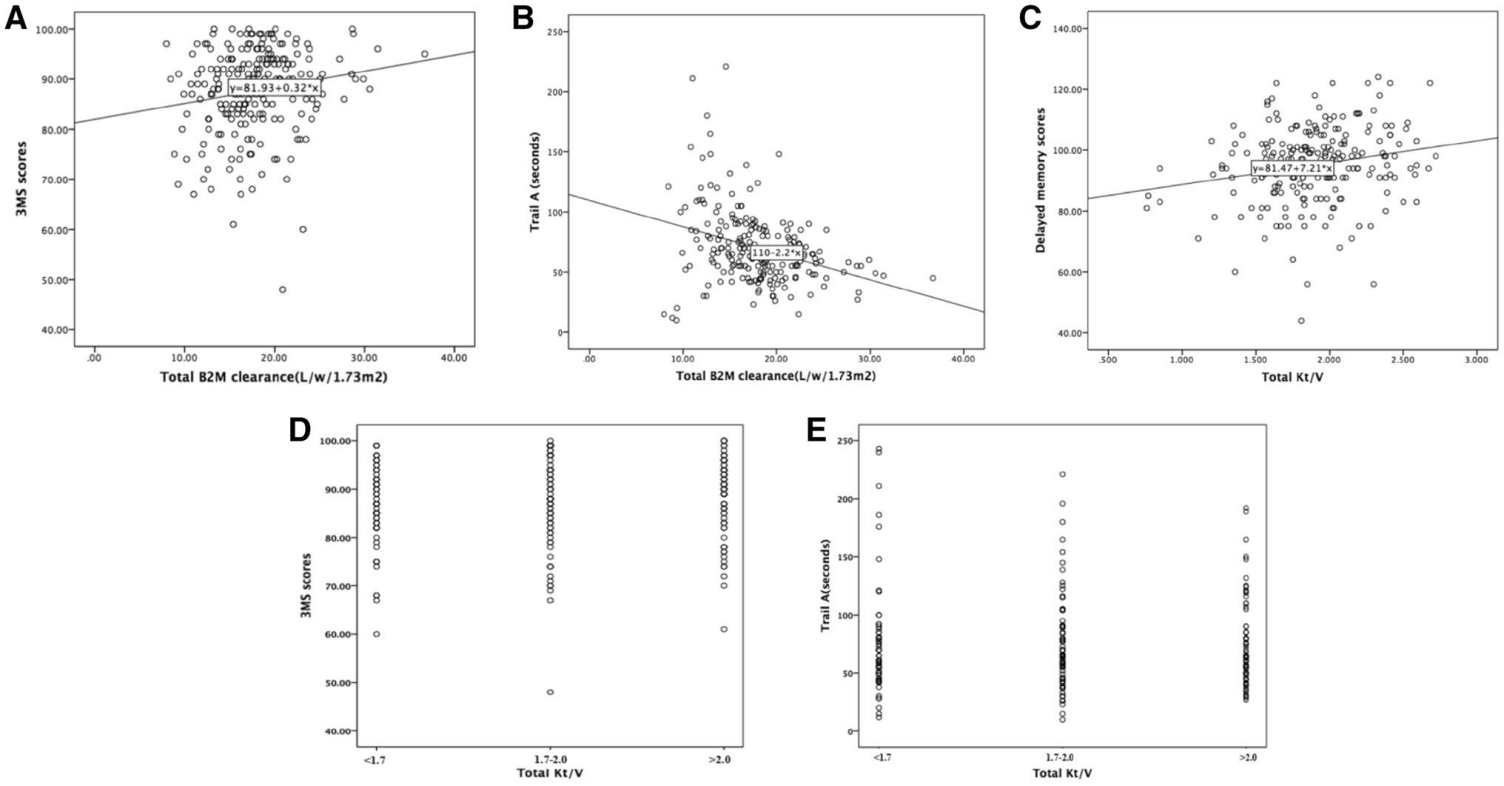
Scatter plot of B2M clearance and KT/V versus cognitive test scores. *3MS* Modified Mini-Mental State Examination, *B2M* beta-2 microglobulin, *Trail-A* trail making test A, *Kt/V* urea clearance per week

Associations between serum concentration of urea, creatinine and B2M, and Kt/V, Ccr and B2M clearance and cognitive function at baseline during 222 patients were examined by multivariate linear regression analyzes respectively. As shown in Table 2, after adjusting for age, dialysis vintage, education, diabetes, serum albumin, hs-CRP, hyponatremia and mean arterial pressure, total Kt/V was only independently associated with delayed memory (*P* = 0.015). Next, total B2M and dialysate B2M clearance was significantly associated with 3MS scores and completion time on trail A test after multivariate adjustment (*P* = 0.018 and 0.016 for 3MS scores, and *P* < 0.001 for trail A). Scatter plots between total B2M clearance and 3MS, total B2M clearance and trail A, total KT/V and delayed memory were shown in Fig. 2. In Fig. 2a and b, we observed that an increase in total B2M clearance was positively associated with a higher 3MS scores and a reduction in completion time of trail A. In Fig. 2c, we found that an increase in total Kt/V was associated with a better score of delayed memory (Fig. 2). Neither total nor renal B2M clearance did correlate to scores for immediate memory, delayed memory, visuospatial skill and language skill. No any relationships were found between serum urea, creatinine and B2M concentration, total, dialysate and renal Ccr, and scores for general and specific cognitive function (*P *> 0.05).

**Table 2:** Associations between the concentration and clearance of small, middle molecules solute and cognitive function at baseline by multivariable linear regression analysis in 222 patients (95% confidence interval for β coefficients as shown)

	3MS score^#^	Trail A, s	Trail B, s	Immediate memory score^#^	Delayed memory score^#^	Visuospatial skill score^#^	Language skill score^#^
Serum urea (mmol/l)	− 0.09 (− 0.30, 0.12)	− 1.43 (− 3.02, 1.57)	− 1.32 (− 3.93, 1.28)	− 0.23 (− 0.67, 0.20)	− 0.14 (− 0.48, 0.21)	0.19 (− 0.41, 0.79)	0.03 (− 0.33, 0.38)
Serum creatinine (μmol/l)	− 0.001 (− 0.005, 0.003)	0.00 (− 0.03, 0.03)	− 0.04 (− 0.09, 0.02)	− 0.006 (− 0.01, 0.002)	− 0.003 (− 0.009, 0.004)	− 0.004 (− 0.02, 0.008)	0.003 (− 0.004, 0.01)
Serum B2M (mg/l)	− 0.14 (− 0.59, 0.33)	1.03(− 2.44, 4.50)	− 0.08 (− 5.73, 5.57)	0.23 (− 0.71, 1.17)	0.31 (− 0.44, 1.05)	− 0.54 (− 0.18, 0.76)	0.12 (− 0.66, 0.89)
Total Kt/V	2.43 (− 0.41, 5.27)	− 1.52 (− 22.7, 19.7)	5.16 (− 29.70, 40.01)	4.19 (− 1.72, 10.1)	6.19 (1.62, 10.8)**	4.83 (− 3.12, 12.8)	− 0.05 (− 4.79, 4.69)
Renal Kt/V	− 0.36 (− 2.84, 2.11)	− 10.5 (− 28.8, 7.84)	2.95 (− 27.49, 33.39)	0.77 (− 4.37, 5.90)	1.08 (− 2.95, 5.11)	6.87 (0.04, 13.7)	− 0.33 (− 4.43, 3.77)
Peritoneal Kt/V	1.56 (− 0.52, 3.65)	6.71 (− 8.84, 22.27)	0.69 (− 24.86, 26.24)	1.70 (− 2.64, 6.04)	2.56 (− 0.84, 5.96)	− 2.33 (− 8.16, 3.49)	0.21 (− 3.26, 3.68)
Total Ccr (l/w/1.73 m^2^)	0.007 (− 0.06, 0.08)	− 0.17 (− 0.69, 0.36)	− 0.07 (− 0.92, 0.79)	0.05 (− 0.09, 0.19)	0.07 (− 0.05, 0.18)	0.16 (− 0.03, 0.36)	− 0.03 (− 0.15, 0.09)
Renal Ccr (l/w/1.73 m^2^)	− 0.01 (− 0.07, 0.04)	− 0.23 (− 0.63, 0.17)	0.05 (− 0.62, 0.71)	− 0.02 (− 0.09, 0.13)	0.01 (− 0.08, 0.10)	0.15 (− 0.001, 0.29)	− 0.02 (− 0.11, 0.07)
Peritoneal Ccr (l/w/1.73 m^2^)	0.04 (− 0.04, 0.13)	0.32 (− 0.30, 0.95)	− 0.20 (− 1.23, 0.83)	0.04 (− 0.14, 0.21)	0.07 (− 0.07, 0.21)	− 0.12 (− 0.36, 0.11)	0.009 (− 0.13, 0.15)
Total B2M clearance (l/w/1.73 m^2^)	0.25 (0.04, 0.46)*	− 2.55 (− 3.86, − 1.23)***	− 1.17 (− 3.88, 1.53)	0.13 (− 0.25, 0.51)	− 0.02 (− 0.31, 0.28)	0.02 (− 0.49, 0.53)	0.06 (− 0.24, 0.37)
Renal B2M clearance (l/w/1.73 m^2^)	− 0.29 (− 0.71, 0.14)	− 1.25 (− 4.40, 1.89)	0.12 (− 5.14, 5.38)	− 0.37 (− 1.25, 0.49)	0.06 (− 0.62, 0.75)	0.59 (− 0.58, 1.78)	0.07 (− 0.63, 0.78)
Peritoneal B2M clearance (l/w/1.73 m^2^)	0.21 (0.02, 0.41)*	− 2.50 (− 3.92, − 1.09)***	− 0.80 (− 3.01, 1.40)	0.26 (− 0.15, 0.66)	− 0.02 (− 0.33, 0.30)	− 0.08 (− 0.63, 0.47)	0.05 (− 0.28, 0.38)

All models were adjusted for age, dialysis vintage, education level, the presence of diabetes, serum albumin, hs-CRP, hyponatremia and mean arterial pressure

*Kt/V* urea clearance per week, *Ccr* creatinine clearance per week, *B2M* beta-2 microglobulin

#The higher scores for 3MS, immediate and delayed memory, language and visuospatial function meant the better performance. The higher score for trails A meant the worse executive function. T scores of immediate and delayed memory, language and visuospatial function were applied in above analyzes

^*^P < 0.05, ^**^P < 0.01, ^***^P < 0.001 for all β coefficients

### Small and middle molecules clearance and the change of cognitive function during the follow-up

Two years later, general and specific cognitive function was repeatedly assessed in 130 patients (Table 3). By comparisons in scores for each test, we observed that 3MS scores significantly increased (*P *= 0.02) and completion time on trail A numerically decreased (*P *= 0.06) after 2 year for the whole cohort.

**Table 3:** Comparisons in cognitive function parameters at baseline and 2-year later (*n* = 130)

	Baseline	2-year later	*P*
3MS (scores)	88.7 ± 7.24	90.0 ± 7.63	0.02*
CI, *n* (%)	16 (12.3%)	20 (15.4%)	0.54
Trail A (seconds)	61 (50–79)	59 (46–80)	0.06
Trail B (seconds)	159 (110–230)	145 (107–192)	0.18
Immediate memory (scores)	76.5 ± 16.7	78.7 ± 17.6	0.09
Delayed memory (scores)	96.7 ± 11.2	97.7 ± 15.2	0.43
Visuospatial skill (scores)	97.2 ± 21.8	98.0 ± 15.1	0.69
Language ability (scores)	96.4 ± 12.9	97.8 ± 11.3	0.14

*3MS* Modified Mini-Mental State Examination, *CI* cognitive impairment

^*^P < 0.05, comparison between baseline and 2-year later

To explore the effect of small and middle molecules clearance on the changing trend of 3MS and trail A test, we further divided our patients into groups according to total Kt/V and B2M clearance respectively (Table 4). For the changing trend of 3MS test, patients with varied Kt/V values did not show a significant difference over 2 years between groups. However, patients with varied total B2M clearance had significantly different trend of 3MS scores even adjusted for baseline 3MS scores and additionally recognized confounders (*P *= 0.033 and 0.027, respectively). Among patients with total B2M clearance > 19.0 l/w/1.73 m^2^, their general cognitive function markedly improved as shown by 3MS (*P *= 0.005), as compared to patients with total B2M clearance < 15.2 l/w/1.73m^2^ or 15.2–19.0 l/w/1.73 m^2^. For the changing trend of scores for executive function, patients with varied values for total Kt/V or B2M clearance did not show any significant differences. However, patients with total B2M clearance > 19.0 l/w/1.73 m^2^ showed a marginally declined completion time on trail A test after 2 year (*P *= 0.05) (Table 5). We also showed the scatter plots to compare the 3MS scores and completion time of trail A between patients categorized by total Kt/V in Fig. 2d and e. There were no differences in these scores between groups with total kt/V < 1.7, 1.7–2.0 and > 2.0 (Fig. 2).

**Table 4:** Associations between small, middle molecules clearance and the change of general cognitive function between groups

	3MS (scores)		*P* for pairs	*P* for trend^#^	*P* for trend*
	Baseline	2-year later			
Total Kt/V				0.92	0.73
< 1.7	87.2 ± 7.85	89.5 ± 7.78	0.12		
1.7–2.0	87.4 ± 7.77	89.1 ± 1.16	0.08		
> 2.0	90.5 ± 6.01	91.1 ± 7.31	0.53		
Total B2M clearance (l/w/1.75 m^2^)				0.033	0.027
< 15.2	88.4 ± 6.92	88.2 ± 8.85	0.89		
15.2–19.0	88.6 ± 7.46	90.6 ± 6.27	0.07		
> 19.0	88.8 ± 7.47	91.2 ± 7.31	0.005		

*Kt/V* urea clearance per week, *B2M* beta-2 microglobulin

*Adjusted for age, dialysis vintage, education level, the presence of diabetes, serum albumin, hs-CRP, hyponatremia, mean arterial pressure and baseline 3MS scores

^#^Adjusted for baseline 3MS scores

**Table 5:** Associations between small, middle molecules clearance and the change of executive function between groups

	Trail A test (seconds)		*P* for pairs	*P* for trend^#^	*P* for trend*
	Baseline	2-year later			
Total Kt/V				0.67	0.11
< 1.7	65 (48, 85)	56 (41, 77)	0.13		
1.7–2.0	65 (45, 92)	63 (48, 80)	0.33		
> 2.0	63 (50, 85)	53 (44, 76)	0.06		
Total B2 M clearance (l/w/1.73 m^2^)				0.41	0.23
< 15.2	66 (55, 91)	60 (48, 85)	0.14		
15.2–19.0	60 (50, 79)	57 (47, 81)	0.74		
> 19.0	58 (48, 73)	55 (44, 78)	0.05		

*Kt/V* urea clearance per week, *B2M* beta-2 microglobulin

*Adjusted for age, dialysis vintage, education level, the presence of diabetes, serum albumin, hs-CRP, hyponatremia, mean arterial pressure and baseline 3MS scores

^#^Adjusted for baseline 3MS scores

Other scores for trail B test, immediate and delayed memory, visuopatial skill and language ability were not significantly different over the time in the whole cohort. The changing trends of trail B test, immediate memory, delayed memory, language and visuospatial function were not found to be different according to the tertiles of total Kt/V and B2M clearance during the follow-up.

## Discussion

This present study revealed that middle solute clearance assessed by total and dialysate B2M clearance, were independently associated with general cognitive function and executive function at baseline. The higher total B2M clearance was also independently predictive of an improvement in general cognitive function after 2 years. Indeed, a recent study reported that the increased concentrations of serum B2M that may predispose to deposition of amyloid in the tissues of HD patients are not associated with a recognizable cognitive deficit [32]. Therefore, we should further explore the association between serum and cerebral B2M concentration, and whether B2M deposits in the brain contributes to cognitive impairment based on human researches. In addition, previous study showed that patients starting from haemodiafiltration 60 ml/min experienced a significantly higher B2M clearance vs HD [33]. The effect of different dialysis modalities on cognitive function should be explored further.

Our data suggested that serum B2M concentration was not associated with cognitive dysfunction at baseline and during follow-up. Although serum B2M have been proved to be a predictor of cardiovascular disease and mortality in dialysis patients [34–37], our findings cannot verify the harmful effect of serum B2M per se in cognitive function. There are several potential causes for this phenomenon. First, previous evidence on the association of B2M and cognitive function are only from animal models instead of human researches. For example, exogenous B2M injected systemically, or locally in the hippocampus, impairs hippocampal-dependent cognitive function and neurogenesis in young mice [38]. The absence of endogenous B2M expression abrogates age-related cognitive decline and enhances neurogenesis in aged mice [38]. Secondly, whether B2M concentration in serum is correlated to B2M deposits in the brain is not determined. One study performed in HD patients indicated that the B2M concentration in the cerebrospinal fluid is maintained under the lower limit of cytotoxicity in the cell culture, which seemed not to support the hypothesis on the harmful effect of serum B2M [39]. Therefore, we should further explore the association between serum and cerebral B2M concentration, and whether B2M deposits in the brain contributes to cognitive impairment based on human researches. It is also of interest to know if B2M accumulation is relevant to abnormalities in cerebral structure such as white matter hyperintensities and subcortical small-vessel disease, as commonly found in the elderly and CKD patients with cognitive decline [40–44].

The negative impact of total and dialysate B2M in the decline in cognitive function needs to be explained cautiously. B2M clearance possibly reflects adequate clearance of similar molecules. We cannot exclude the possibility that other middle-molecule or protein-bound toxics contribute to the association between B2M clearance and declined cognitive function. In addition, a higher B2M clearance is calculated from a larger amount of dialysis dose, higher ultrafiltration, or a smaller body size. All above are potential confounders need to be considered. Despite that, these clues to a close relationship between B2M clearance and the improvement in cognitive function, suggesting the feasibility of trials on enhancing middle-molecule clearance as an interventional strategy for the treatment of CI. The only one randomized controlled study on frequent hemodialysis to augment removal of uremic solute, did not show positive effects as compared with conventional three times per week [17]. Of note, the dialysis frequency was increased for intervention group while the length of dialysis per session shortened and dialyzer unchanged, which is not helpful to increase middle-molecule solute clearance. Accordingly, a study designed for improving both middle- and small-molecule clearance by increasing both times and dwell time of PD exchange [45] should be performed to observe their potential benefits on cognitive function in PD patients.

As shown in our data, renal Kt/V, Ccr and B2M clearance were not significantly associated with the baseline and subsequent cognitive function. This finding could not deny that residual renal function may play its role in the maintenance of cognitive function. The mean levels of renal Kt/V, Ccr and B2M were only 0.52, 22.7 l/w/1.73 m^2^ and 3.3 l/w/1.73 m^2^, respectively in our patients, which probably is not meaningful to impact the patient outcome. Also, the longitudinal changes of cognitive function according to small molecule solute clearance have not been found, which might be due to the small sample size and short observation period.

Of note, we found that general cognitive function assessed by 3MS was significantly improved despite that all specific items of cognition did not change after 2 years. This finding is paradoxical to previous studies showing the cognitive function would get worse over the time on the dialysis. This phenomenon might be first explained by the underestimation of decline in cognitive function due to case bias from drop-outs (survivor bias). Other possible explanations for this phenomenon might be that participants included in this study had higher serum albumin levels (38.9 ± 3.2 g/l) as compared to those in previous studies [46]. Serum albumin level was an independently favorable factor to maintain the cognitive function as shown in our multicenter prospective cohort study [47]. A recent meta-analysis study indicated that PD might be superior in preserving the cognitive functions and decreasing the risk of dementia compared with hemodialysis from totally 15 cohort or cross-sectional studies [48]. The authors explained that the continuity and stability of PD therapy is beneficial for restoring the cognitive function. However, we still cannot draw the conclusion on the practice effect of PD on the improvement of cognitive function except that further interventional studies on the dialysis modality and cognitive function provide more robust evidence.

Of interest, we found the association between total B2M clearance and trail A rather than trail B. The difference in two test for screening executive function should be discussed. The connection mode for trail A was that arabic numeral 1 connected to arabic numeral 2, arabic numeral 2 connected to arabic numeral 3(1–2–3),etc. On the other hand, connection mode for trail B was that arabic numeral 1 connected to the letter A, and the letter A was connected to arabic numeral 2, arabic numeral 2 was connected to letter B (1-A–2-B), etc. Our Chinese PD participants are generally not familiar with the letter due to their old ages. Trail A might be more sensitive to reflect executive dysfunction than trail B.

Several advantages are described as below. There are few data indicating the association of small- and middle-molecule solute clearance and cognitive function in dialysis patients. A wide range of cognitive tests encompassing a broad spectrum of cognitive domains such as execution function, memory, visuospatial and language function were examined with general cognitive assessments.

We are also aware of the limitations of this study. First, due to the nature of an observational study, whether B2M clearance is a pathogenic factor or solely a risk biomarker cannot be determined. A randomized controlled study to explore whether cognitive function is improved by adjusting dialysis prescription to increase clearances of small and middle solute would be useful. Second, potential co-pathogenesis behind B2M clearance and cognitive function needs to be further explored. The single-time measurement of B2M clearance and absence of large-solute toxins information are additional limitations. Almost 40% of participants were not included into the second assessment, which could underestimate the declined trend of cognitive function since drop-outs tended to be sicker at baseline. The clinical differences in 3MS scores and trail A test over 2 years in patients with higher total B2M clearance still need to be determined despite of its statistic differences.

In summary, based on this longitudinal cohort dataset, we indicated that middle solute clearance is closely associated with baseline general cognitive and executive function, and also subsequent cognitive change. Accordingly, regular screening for small-, middle- and large-solute toxins clearance in a larger sample with the longer observation period, is required to understand their roles in the decline of cognitive function among dialysis patients. A novel direction for further research is proposed, exploring the potential effects of adjusting dialysis prescription to increase clearance of small and middle solute clearances, avoiding of cognitive dysfunction.

## Electronic supplementary material

Below is the link to the electronic supplementary material.

Supplementary material 1 (TIFF 2700 kb)

Supplementary material 2 (TIFF 2700 kb)

Supplementary material 3 (TIFF 2700 kb)

Supplementary material 4 (TIFF 2700 kb)

Supplementary material 5 (TIFF 2700 kb)
