# Supplementary figures and images for: Associations between small and middle molecules clearance and the change of cognitive function in peritoneal dialysis
Source: J Nephrol. 2019 Oct 23;33(4):839–48. doi: 10.1007/s40620-019-00661-8 (PMC7381472; doi:10.1007/s40620-019-00661-8)

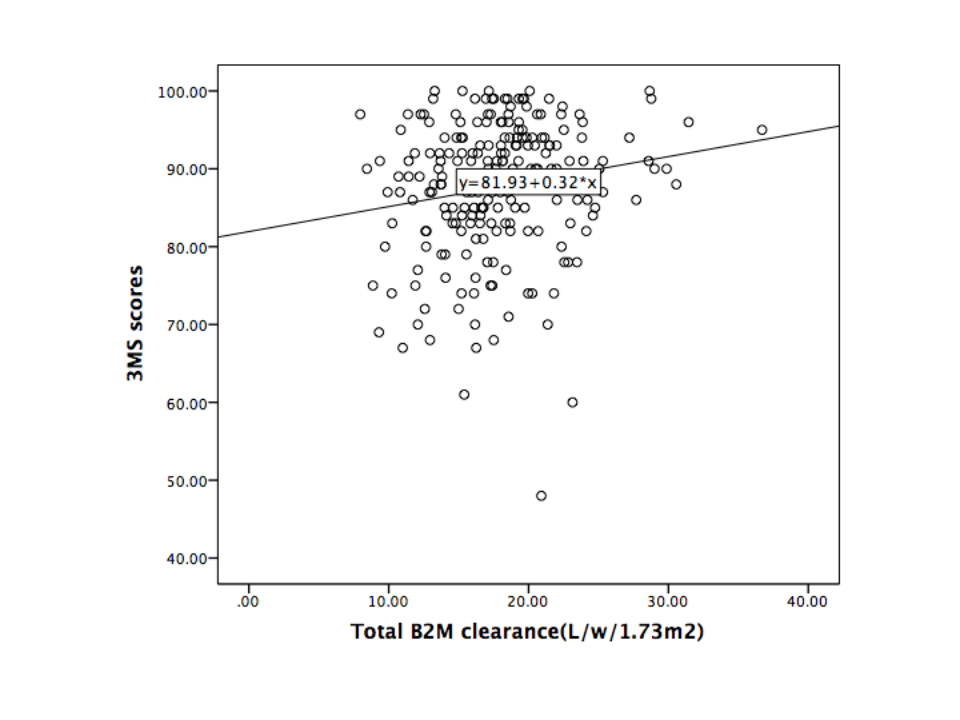

Supplement: Supplementary file 1 — Supplementary material 1 (TIFF 2700 kb) [file 40620_2019_661_MOESM1_ESM.tif]

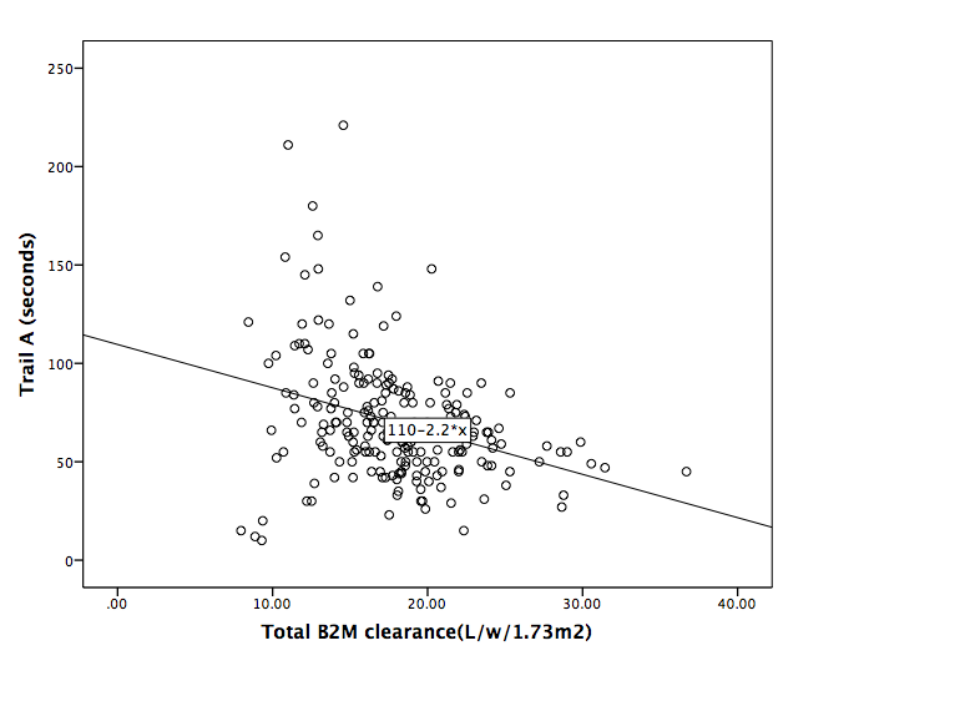

Supplement: Supplementary file 2 — Supplementary material 2 (TIFF 2700 kb) [file 40620_2019_661_MOESM2_ESM.tif]

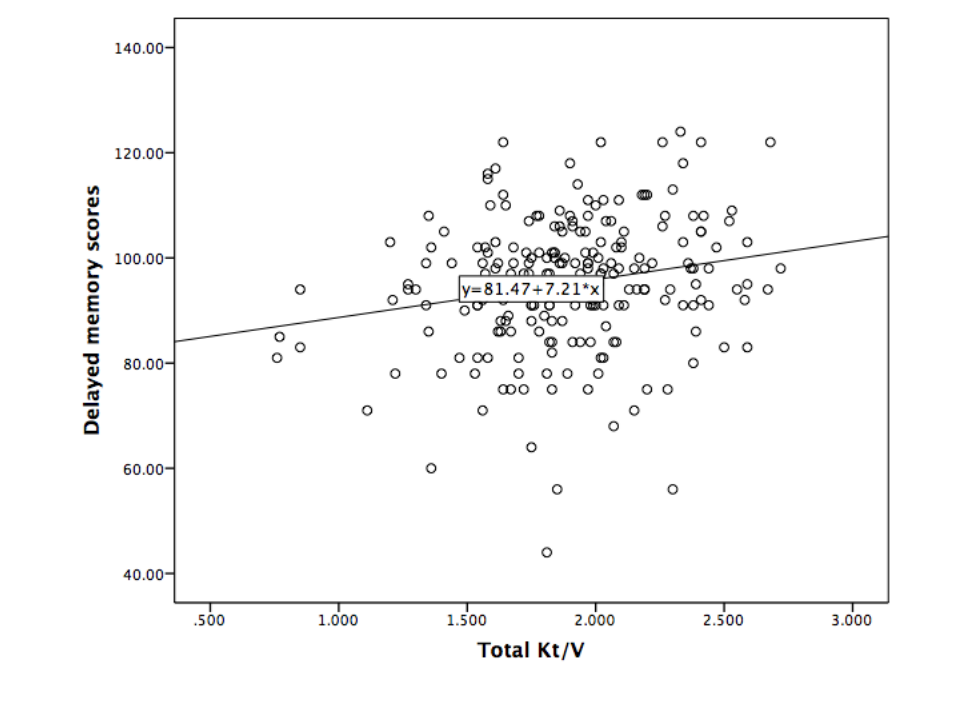

Supplement: Supplementary file 3 — Supplementary material 3 (TIFF 2700 kb) [file 40620_2019_661_MOESM3_ESM.tif]

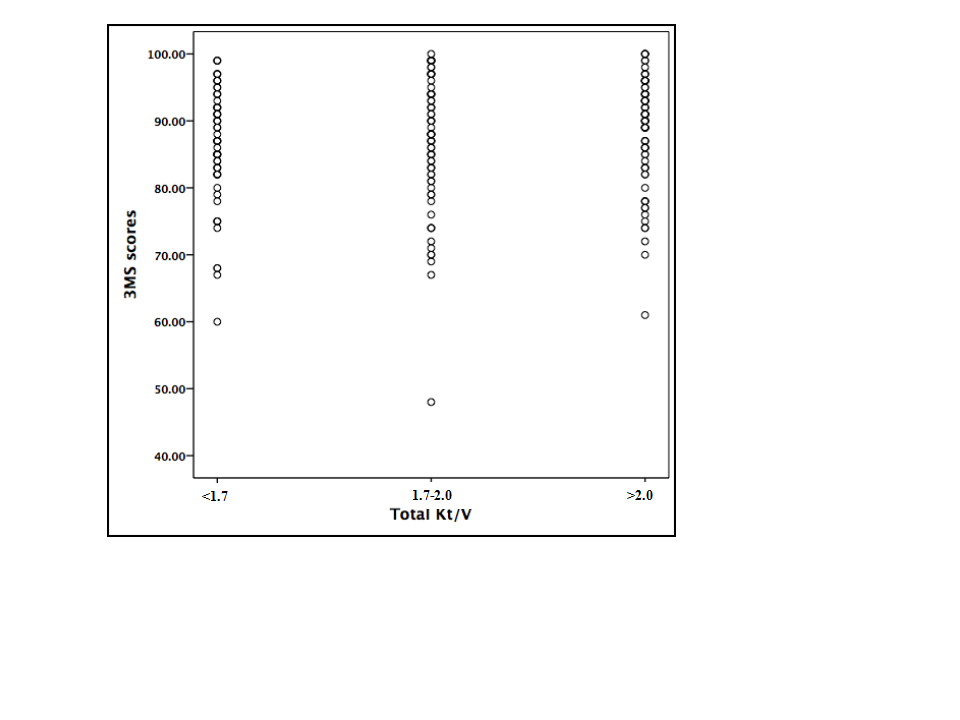

Supplement: Supplementary file 4 — Supplementary material 4 (TIFF 2700 kb) [file 40620_2019_661_MOESM4_ESM.tif]

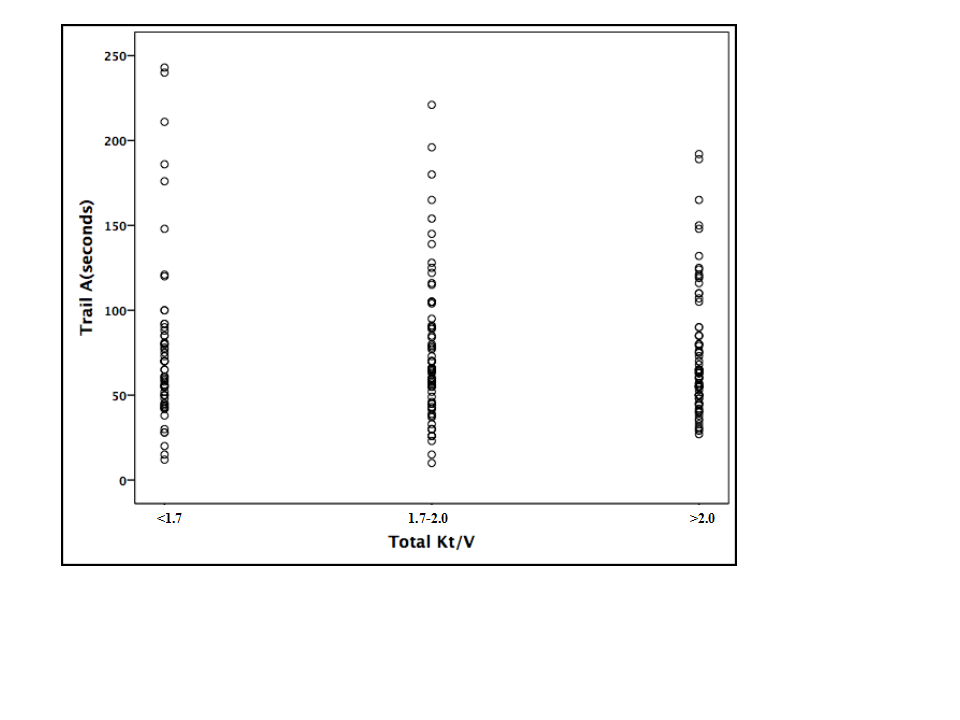

Supplement: Supplementary file 5 — Supplementary material 5 (TIFF 2700 kb) [file 40620_2019_661_MOESM5_ESM.tif]
